# Meaningful orientations and asthenia in pregnant women and young mothers during the first COVID-19 pandemic wave

**DOI:** 10.1192/j.eurpsy.2022.1244

**Published:** 2022-09-01

**Authors:** V. Titova, D. Ivanov, Y. Aleksandrovich, I. Gorkovaya, V. Rozhdestvenskiy

**Affiliations:** Saint Petersburg State Pediatric Medical University, Department Of Psychosomatics And Psychotherapy, Saint Petersburg, Russian Federation

## Abstract

**Introduction:**

Pregnancy and childcare are naturally stressful for women, often accompanied by the asthenic syndrome. In a pandemic situation, this type of stress may be potentiated by external conditions.

**Objectives:**

The study aimed to investigate the life-state orientations and asthenia levels of pregnant women and young mothers in the context of a pandemic. We also analyzed the correlations between the life-state orientations and the different types of asthenias.

**Methods:**

Data collection was carried out in June 2020 using a Google form that we developed. Pregnant women and young mothers with children under seven years of age participated in the study with 47 respondents. We used the Purpose-in-Life Test to investigate the meaningful orientations and the MFI-20 to determine the level of asthenia. Both questionnaires were adapted for use in Russia.

**Results:**

We found that the mean overall MFI-20 score (M = 58.0±5.9) exceeded the mean values in our sample, indicating the presence of the asthenic syndrome. Physical asthenia (M = 12.9±1.4) and decreased activity (M = 12.0±1.7) were the strongest, with the lowest score on the general asthenia scale (M = 10.6±1.8). Correlation analysis showed that all components of meaningful orientations had multiple positive correlations with different types of asthenias, and the overall asthenia score was 100 % related to life meaningfulness (p < 0.01).

**Conclusions:**

Pregnant women and young mothers are at risk for asthenia in the COVID-19 pandemic. This is obviously due to many responsibilities of mothers raising children.

**Disclosure:**

No significant relationships.

